# Domain Growth in Polycrystalline Graphene

**DOI:** 10.3390/nano13243127

**Published:** 2023-12-13

**Authors:** Zihua Liu, Debabrata Panja, Gerard T. Barkema

**Affiliations:** Department of Information and Computing Sciences, Utrecht University, 3584 CC Utrecht, The Netherlands; z.liu1@uu.nl (Z.L.);

**Keywords:** polycrystalline graphene, domain growth, grain boundary, Monte Carlo dynamics, disordered materials

## Abstract

Graphene is a two-dimensional carbon allotrope which exhibits exceptional properties, making it highly suitable for a wide range of applications. Practical graphene fabrication often yields a polycrystalline structure with many inherent defects, which significantly influence its performance. In this study, we utilize a Monte Carlo approach based on the optimized Wooten, Winer and Weaire (WWW) algorithm to simulate the crystalline domain coarsening process of polycrystalline graphene. Our sample configurations show excellent agreement with experimental data. We conduct statistical analyses of the bond and angle distribution, temporal evolution of the defect distribution, and spatial correlation of the lattice orientation that follows a stretched exponential distribution. Furthermore, we thoroughly investigate the diffusion behavior of defects and find that the changes in domain size follow a power-law distribution. We briefly discuss the possible connections of these results to (and differences from) domain growth processes in other statistical models, such as the Ising dynamics. We also examine the impact of buckling of polycrystalline graphene on the crystallization rate under substrate effects. Our findings may offer valuable guidance and insights for both theoretical investigations and experimental advancements.

## 1. Introduction

**Figure 1:**
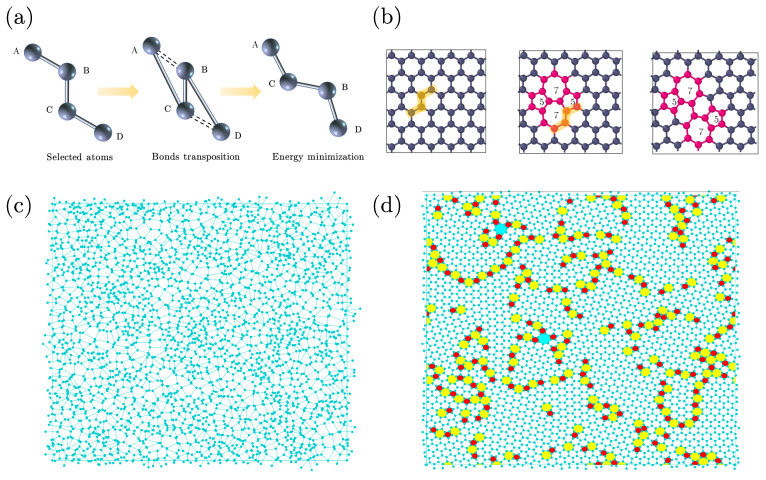
(**a**) Elementary move in the structural evolution of polycrystalline graphene, also known as a bond transposition. In a string of four carbon atoms A-B-C-D, bonds A-B and C-D are replaced by bonds A-C and B-D, leaving the central bond, B-C, untouched. (**b**) Left panel: if bond transposition occurs in crystalline graphene, it results in two oppositely oriented pairs of 5–7 rings. Right panel: if bond transposition occurs in the immediate vicinity of a 5–7 pair, it effectively displaces sideways.The atoms marked in orange are selected for bond transposition. (**c**) Visualization of an initial sample, created from a Voronoi network as described in the text. We note that the network is disordered and homogeneous, with at most tiny crystalline regions. (**d**) Same network after structural relaxation with $9\times 10^{4}$ proposed bond transitions, when crystalline regions have appeared. In this figure, 5-, 7- and 8-fold rings are marked in different colors.

Graphene is a two-dimensional (2D) material in which carbon atoms are organized in the structure of a honeycomb lattice. It exhibits a wide range of appealing properties in comparison to more conventional materials, including exceptionally high strength and toughness [1,2,3,4,5,6], remarkable thermal conductivity [7,8,9,10,11], and outstanding electrical conductivity [12]. As a result, use of graphene-based devices has witnessed a substantial surge in recent years [13,14,15,16]. Graphene can be fabricated experimentally through different methods, such as chemical vapor deposition (CVD) [17,18,19,20], epitaxial growth on silicon carbide [21,22], and liquid-phase exfoliation [23,24]. However, graphene produced using these techniques typically exists in a polycrystalline form, which means that the structure consists of many crystalline domains, each with its own lattice orientation. Neighboring domains are separated by strings of defects, usually five-fold and seven-fold rings. A sample of polycrystalline graphene is depicted in Figure 1d. Polycrystalline graphene grown on substrates often exhibits out-of-plane buckling near defects, a phenomenon that has been reported in numerous experiments and simulations [25,26,27]. Buckling can significantly affect the properties of graphene; however, its impact on the growth process is still less understood.

The structure of polycrystalline graphene is not stationary in time. Changes in the bonded structure occur all the time via so-called bond translocations. If such bond translocation occurs in the middle of a crystalline region, four six-fold rings evolve into two five- and two sevenfold rings (middle panel of Figure 1b), a structure known as a Stone–Wales defect. Occasionally arising Stone–Wales defects in otherwise crystalline graphene tend not to last, and in due time, the crystalline structure is restored. If a bond translocation occurs in the immediate vicinity of a 5- and 7-fold ring, the result is that this 5–7 pair is actually displaced sideways (right panel of Figure 1b). Via this mechanism, the domain walls separating the crystalline regions, consisting of alternating strings of five- and sevenfold rings, can actually wander.

The global effect of this wandering of the domain walls is coarsening or domain growth: bigger domains tend to grow at the expense of smaller ones, because of energetic considerations, and the density of domains decreases in time.

Here, we study the domain growth process in graphene using computer simulations. First, in order to understand the force that drives the coarsening process, we study the energetics of polycrystalline graphene: in particular, we show that the total energy of the system increases monotonically with the number of 5- and 7-fold rings in a more or less linear fashion. Next, we study the evolution in time of defect density, spatial correlation of the lattice orientation and the average domain size. We find that the defect density scales as $t^{-1/3}$ in flat polycrystalline graphene, the spatial correlation of the lattice orientation is well fitted by a stretched exponential function, and the average size of the domains grows like $t^{1/6}$. We discuss similarities to the domain growth process (so-called Ostwald ripening) in the Ising model. We also investigate the influence of buckling on the coarsening process and find that the buckling of polycrystalline graphene slows it down. This implies that graphene samples with better crystallinity are best produced if the graphene is kept as flat as possible by a substrate.

This paper is organized as follows. First, in Section 2, we describe our model for graphene, including its dynamics. In Section 3, we validate our model, and present that the structures resulting from simulations are in good agreement with experimental data. Next, in Section 4, we present a statistical analysis of the spread in bond lengths and bond angles, structural disorder and defect density as a function of time. We also present an extensive study of lattice orientations, both in its spatial distribution and its dynamics. In Section 4.2, we analyze the diffusive behavior of defects and the separation of crystal phases. In Section 4.3, we discuss the influence of binding to the substrate for buckled polycrystalline graphene. We conclude the paper with a summary in Section 5.

## 2. Model

The overwhelming majority of carbon atoms in graphene are covalently bonded to three neighboring atoms; undercoordinated carbon atoms do exist, but at a density which is so low that it can be safely neglected. In this work, we use the recently developed semiempirical energy potential which has the following form [28]:

(1) $$\begin{array}{c} E=\frac{3}{16}\frac{\alpha }{d^{2}}\sum_{i,j}^{}{\left(r_{ij}^{2}-d^{2}\right)}^{2}+\frac{3}{8}\beta d^{2}\sum_{j,i,k}^{}{\left(\theta_{jik}-\frac{2\pi }{3}\right)}^{2}+\gamma \sum_{i,jkl}^{}r_{i,jkl}^{2}. \end{array}$$

Here, $r_{ij}$ is the distance between two bonded atoms *i* and *j*, $\theta_{jik}$ is the angle between the two bonds connecting atom *i* to atoms *j* and *k* and $r_{i,jkl}$ is the out-of-plane distance from atom *i* to the plane through the three atoms, *j*, *k* and *l*. Parameter α is chosen as 26.060 eV/Å${}^{2}$ to control bond stretching and is fitted to the bulk modulus. Parameter β=5.511 eV/Å${}^{2}$ governs bond shearing and is fitted to the shear modulus. The parameter γ=0.517 eV/Å${}^{2}$ describes the stability of the graphene sheet against buckling; we note that this third out-of-plane term is zero in perfectly flat graphene (2D simulations). d=1.420 Å is the ideal bond length for pure graphene. All these parameters are obtained by fitting to density functional theory (DFT) calculations [28]. We note that the elastic potential strictly requires the bond list where each atom is bonded to exactly three atoms; the number of bonds equals, therefore, 3N/2, in which *N* is the number of atoms. This potential enables one to efficiently estimate the energies of the relatively stable configurations encountered in our simulations of graphene coarsening.

Simulations of covalently bonded materials are typically slow and computationally expensive; their high stability causes the relevant experimental time scales to be well beyond those accessible by standard molecular dynamics simulations. Here, we employ a relatively simple and accurate model for dynamics of polycrystalline graphene, which was initially applied for generating silicon samples with realistic structures. The model constructs atomic configurations generated by the evolution of a continuous random network (CRN) via bond transpositions, which is a well-established and widely used method to generate realistic atomic configurations of carbon/silicon materials. More specifically, we use the algorithm introduced in ref. [29], an improved version of the original method of Wooten, Winer and Weaire (WWW) [30]. The improved bond transposition procedure consists of the following sequential steps: (1) constructing a comprehensive list of bonds in the current sample configuration; (2) randomly selecting four connected atoms (ABCD); (3) breaking the bonds between AB and CD and forming new bonds between AC and BD, as shown in Figure 1a; (4) performing global energy minimization and comparing energy $E_{a}$ after the bond switch with a predefined energy threshold, defined as

(2) $$\begin{array}{c} E_{t}=E_{b}-k_{B}T\ln\left(s\right), \end{array}$$

where $k_{B}$ is Boltzmann constant, *T* is temperature, $E_{b}$ is the energy before the bond transposition and *s* is a uniform random number between 0 and 1. If energy $E_{a}$ after the bond transposition is less then $E_{t}$, the proposed change is accepted; otherwise, it is rejected, and the atoms and the bond list are restored.

To accelerate the evolution program, we first relax only the atoms in the near vicinity of the bond transposition, bringing the total energy down to $E_{l}$. We then estimate energy $E_{a}$ after global relaxation (without performing the global minimization), employing a local energy criterion in terms of the linear relationship between local minimum energy and the total remaining forces ${\left|F\right|}^{2}$:

(3) $$\begin{array}{c} E_{a}\approx E_{l}-c_{f}{\left|F\right|}^{2}. \end{array}$$

Here, $c_{f}$ is a linear factor obtained from simulations. In our recent work [31], we found that the performance of the local decision depends on the set of atoms allowed movement during the local relaxation; for this, a shell of atoms was selected with the shortest-path distance *l* from the atoms involved in the bond transpositions. In the simulations discussed in the paper, *l* and $c_{f}$ are chosen as 3 and $6\times 10^{-3}$ $s^{2}u^{-1}$ to achieve the best performance, respectively. We note that quantity $c_{f}$ is expressed in units of seconds squared over the atomic mass unit.

The minimization approach exerted in our simulations is the so-called fast inertial relaxation engine (FIRE) algorithm, in which parameters corresponding to ref. [32] are set as $N_{min}=5$, $f_{inc}=1.1$, $f_{dec}=0.5$, $\alpha_{start}=0.1$ and $f_{\alpha }=0.99$. Other custom parameters here are set to be $\Delta t_{MD}=0.03$ and $\Delta t_{max}∼10\Delta t_{MD}$. The velocity Verlet method is chosen for the integration in time.

The domain growth presented in this paper consists of Monte Carlo (MC) dynamics, comprising a sequence of proposed bond transpositions described above. The time scale in this MC dynamics can be related to standard molecular dynamics method (MD). Within our MC dynamics, the probability that a specific bond transposition is proposed in one MC unit of time is 2/(3∗2∗2∗N)=1/(6N). Here, factors 1/N, 1/3, 1/2 and 1/2, respectively, arise from selecting a random atom (out of *N* total atoms), then one of its three neighbors, next twice one of the two remaining neighbors, and the factor of 2 comes from the possibility to generate the same string of atoms from two different ends. The acceptance probability is then exp(−βΔE), where ΔE is the energy difference between the initial and final states. Thus, the rate of structural changes in the sample is (1/6N)exp(−βΔE). In molecular dynamics (MD), the rate would be νexp(−βB) where ν is the attempt frequency, often found to be around $10^{-12}$ s${}^{-1}$ = 1 ps${}^{-1}$, and *B* is the energy barrier between the initial and final states. Energy barrier *B* is roughly equal to the sum of the energy for breaking a single bond and, if positive, energy change ΔE.

Figure 1c,d demonstrate the evolution process of polycrystalline graphene samples. Figure 1c presents a Voronoi diagram with a random structure, providing an initial disordered state.We note that this initial state merely provides a homogeneous disordered network without orientational bias, and does not reflect any practical physical process. The construction of the Voronoi diagram involves several steps: (1) randomly choose a set of points within a simulation box; (2) for each seed point, determine its region, i.e., the set of points which are nearer to it, than to another seed point; (3) construct the boundaries of the Voronoi cells, which are formed by the perpendicular bisectors of the lines connecting neighboring seed points; (4) these boundaries are considered the covalent bonds, and the positions where three of these meet are considered as “atom”. Each “atom” within the Voronoi diagram is strictly limited to having three neighbors, and periodic boundary conditions (PBCs) are applied to ensure a constant number of atoms (*N*) and bonds (3*N*/2) within the simulation box. Figure 1d shows the evolution of a polycrystalline graphene structure with a defined defect density achieved by implementing $9\times 10^{4}$ proposed bond transpositions. The nanocrystalline domains with distinct crystal orientations are separated by domain walls consisting mainly of 5- and 7-fold rings. Further, individual defect islands emerge within the crystalline domains.

## 3. Model Validation

The model was first introduced in ref. [28]; it is based on Kirkwood’s potential [33]. This potential has been used, for instance, for studying the structural dynamics of single-layer polycrystalline graphene [34], for studying the long-range relaxation of structural defects [28], for probing crystallinity of graphene samples via their vibrational spectrum [35] and for the study of the discontinuous evolution of the structure of stretching polycrystalline graphene [31].

Crystalline graphene is a 2D material, but as soon as the structure has defects—in particular if it is polycrystalline—the carbon atoms tend to relief stress by buckling, i.e., displace with respect to each other in the out-of-plane direction. For free-floating graphene in vacuum, the buckling can have an amplitude of many angstroms, while for graphene attached to a substrate, the amplitude of the buckling away from the substrate is suppressed significantly. In the first part of our simulations, we confine graphene to a 2D plane without any buckling; further on, we relax the constraint to the plane and allow for buckling.

**Figure 2:**
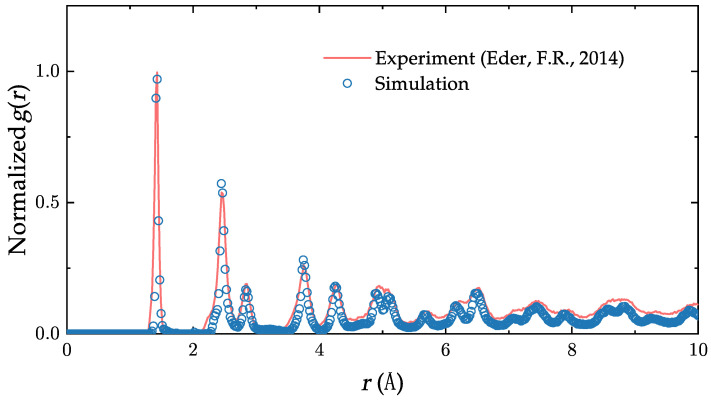
Comparison of the normalized radial distribution function g(r) of our generated sample and experiment at comparable defect density. The two curves match very well, up to about the first ten peaks [37].

In order to ensure the validity of the obtained samples, we employ the radial distribution function (RDF) as defined in Equation (4), which characterizes the average spatial distribution of particles in a system. The RDF is defined as [36]

(4) $$\begin{array}{c} g\left(r\right)=\frac{\lim_{\Delta r\to 0}\frac{N_{r}}{\pi {\left(r+\Delta r\right)}^{2}-\pi r^{2}}}{\rho }, \end{array}$$

where *r* is the radial distance from reference particle, ρ is the average atom density and $N_{r}$ is the number of atoms between *r* and r+Δr. Starting from the initial configuration (Figure 1c), we let the sample evolve in time. We then compare, in Figure 2, the normalized radial distribution function of the samples (Equation (4)) on the 2D plane when the defect density reaches the same value (∼20%) as in the experiment of Eder et al. [37] (The defect density is defined as the ratio of non-hexagonal rings to the total number of rings). The comparison reveals an excellent simulation–experiment agreement. We also note that the simulated samples we used have a long-range disorder similar to that of the ones observed in real polycrystalline graphene.

## 4. Results

### 4.1. Domain Growth in Flat Polycrystalline Graphene

**Figure 3:**
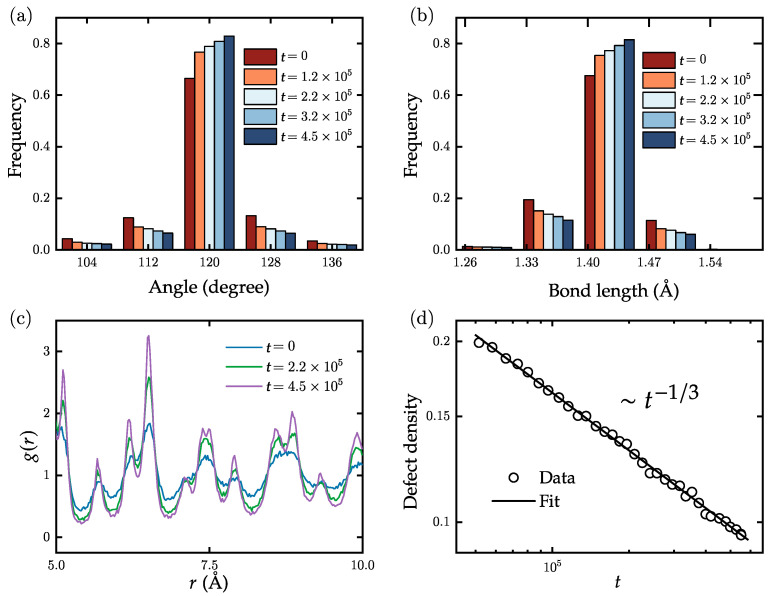
Time evolution of the distribution of (**a**) the bond angles and (**b**) the bond lengths in planar samples of graphene. With increasing simulation times, both distributions become narrower. (**c**) Time evolution of the radial distribution function. With increasing simulation time, the peaks at longer distance become increasingly pronounced. (**d**) Density of defects (5 and 7 rings) as a function of simulation time. The decay can be well fitted by a power-law decay $t^{-1/3}$ (solid line).

For studying domain growth of realistic polycrystalline graphene samples, at t=0, we start with one consisting of 9800 atoms and a defect density of ∼20%. We then evolve it for $4.5\times 10^{5}$ Monte Carlo steps (MCS) under weak pressure and quench to 3000 K, a temperature significantly below the melting temperature of polycrystalline graphene. To improve our statistics, all statistical data presented in the paper are obtained by repeating the evolution process 50 times using different random number seeds. The simulations are performed on an Intel i7-9700 CPU (manufactured by Intel, sourced in Utrecht, The Netherlands), with an average runtime of approximately 0.02 s per MCS. Figure 3a,b display the distributions of bond angles and bond lengths for different times, respectively. We note here that in flat polycrystalline graphene, the third term in the potential function (Equation (1)) related to dihedral angles can be neglected due to the absence of out-of-plane forces. Consequently, the bond angles gradually approach the ideal value of 120${}^{\circ }$, while the bond lengths tend to converge to 1.42 Å. Figure 3c illustrates the time-dependent changes in the RDF in the range of 5 Å to 10 Å. With increasing time, distinct peaks of the RDF appear at multiple positions, indicating the gradual appearance of longer-ranged order and an increase in the domain area. Figure 3d displays the power-law behavior of the defect density as a function of time, with an exponent of −0.330±0.002. Based on this result, we speculate that the exponent for defect density decrease under ideal conditions (adequate statistical sampling) is −1/3 at T=3000 K. Equation (2) indicates that temperature can affect the evolution by either increasing or decreasing the acceptance rate.

**Figure 4:**
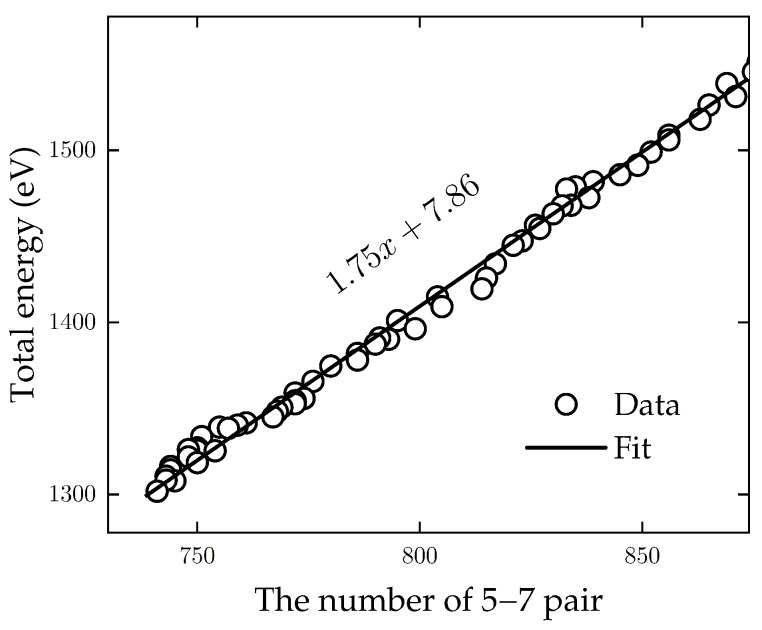
Total energy as a function of the number of defects (pairs of 5 and 7 rings) in planar graphene. The data can be well fitted with a linear relation: E=1.75x+7.86. As a reference, a single SW defect consists of two such pairs and would thus correspond to a defect formation energy of 3.5 eV.

Figure 4 shows the linear relationship between the total energy and the number of 5–7 pairs; it can be linearly fitted by f(x)=1.75x+7.86. As a reference, this corresponds to the formation energy of a single Stone–Wales (SW) defect in the flat polycrystalline graphene by nearly 3.5 eV [28], as each SW defect consists of two 5–7 pairs.

During the growth process of polycrystalline graphene, it is common to observe the formation of domains with different lattice orientations. The lattice orientation of these domains is complex and influenced by various factors, such as the motion of individual defects, the alignment of domain boundaries, and external pressure. These factors can exert torques to the domains, leading to a certain degree of lattice rotation within the domains. For graphene, the range of lattice orientation is $-30^{\circ }$ to $30^{\circ }$, with positive values indicating orientations corresponding to rotations around the *z*-axis in the positive direction and negative values indicating orientations pointing towards the negative direction of rotation around the *z*-axis. Identification of the crystal orientation in disordered 2D materials relies on descriptors to quantify the local order in atomic systems [38,39,40,41]. Here, we apply polyhedral template matching (PTM) to identify the lattice orientation of atoms in polycrystalline graphene [42]. This method enables classification of structures according to the topology of the local atomic environment, without any ambiguity in the classification, and with greater reliability than, e.g., common neighbor analysis in the presence of thermal fluctuations. It is important to note the custom parameter root-mean squared deviation (RMSD); a higher RMSD cutoff leads to more identifications (and fewer defect atoms), though possibly at the expense of false positives. A lower RMSD cutoff results in fewer structural identifications (and more defect atoms and greater sensitivity to perturbations of the lattice), though possibly at the expense of false negatives. The RMSD is set to 0.1 in our simulations to achieve optimal identification results. With this setting, the hexagonal lattice structure and defects can be identified relatively accurately. However, the identification performance for defects is not as robust as the ring identification algorithm used in the previous text, which can identify non-hexagonal ring defects with 100% accuracy.

**Figure 5:**
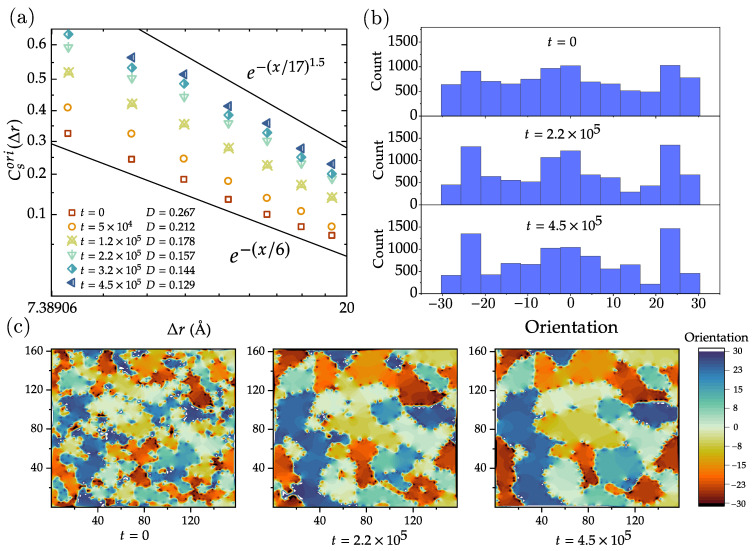
Analysis of the orientation of the hexagons in the lattice —10 −10−10 structure. (**a**) Normalized correlation function $C_{s}^{\mathrm{ori}}$ of the orientations as defined in Equation (5), as a function of distance *r*, for various times. The data show a linear trend if $\left(-\ln\left(C_{s}^{\mathrm{ori}}\right)\right)$ is plotted as a function of distance *r* in a double-logarithmic plot, indicating that correlation function $C_{s}^{\mathrm{ori}}$ decays as a stretched exponential. (**b**) Histogram of the hexagon orientations at different times. While the crystalline regions grow in time, these histograms become increasingly rugged. (**c**) Evolution of the maps of hexagon orientations. Some regions grow (while conserving their orientation) at the expense of other regions that shrink and sometimes disappear.

In order to investigate the spatial distribution of the lattice orientation, we define the normalized spatial correlation of the lattice orientation $C_{s}^{\mathrm{ori}}$ below:

(5) $$\begin{array}{c} C_{s}^{\mathrm{ori}}\left(\Delta r\right)=\frac{\langle o_{i}\times o_{j}\rangle }{\langle o_{i}^{2}\rangle }, \end{array}$$

where $o_{i}$ and $o_{j}$ are the orientation of atoms *i* and *j*, respectively, ${\vec{r}}_{i}$ and ${\vec{r}}_{j}$ are the corresponding position, and with fixed distance $\Delta r=\left|{\vec{r}}_{i}-{\vec{r}}_{j}\right|$. Figure 5a shows the variation of $C_{s}^{\mathrm{ori}}$ as a function of Δr at different times *t* and corresponding defect density *D*. The vertical axis shows $\ln[-\ln\left(C_{s}^{\mathrm{ori}}\right)]$, while the horizontal axis is logarithmically scaled. The data exhibit a straight decay pattern in the figure, suggesting a trend that follows stretched exponential decay with a form like $C_{s}^{\mathrm{ori}}∼e^{-{\left(\Delta r/b\right)}^{c}}$. Due to the limitations of sample size and the effects of periodic boundary conditions, there is a significant amount of noise present on spatial scales larger than 20 Å. As a result, it becomes difficult to present the spatial correlation lattice orientation at larger scales. As shown in Figure 5a, the reference lines indicate that an anomalous exponent *c* is observed in the range from 1 to 1.5.

The histogram distribution plots in Figure 5b illustrate the quantitative analysis of lattice orientations at three different times. Evidently, there is a symmetry around zero orientation, indicating that the polycrystalline graphene can be regarded as a binary mixture composed of two types of regions: those with orientations greater than zero degrees and those with orientations lesser than zero degrees, in equal proportions. The average size of the binary mixture corresponds to the intersection between the correlation curve and the *x*-axis in Figure 5a. In the histogram, three prominent peaks are observed in the intervals ($-30^{\circ }$)–($-20^{\circ }$), ($-10^{\circ }$)–($10^{\circ }$), and ($20^{\circ }$)–($30^{\circ }$), suggesting a higher concentration of atomic orientations within these ranges. Further, we observe that as time progresses from t=0 to $t=4.5\times 10^{5}$ in terms of MCS, the intensity of these peaks increases, which is also in line with the lattice orientation distribution map shown in Figure 5c. There is a trend suggesting that smaller regions with the same orientation have a higher tendency to merge into larger regions, and regions with similar orientations are more prone to fusion.

### 4.2. Dynamics of Crystal Phases

In the samples of polycrystalline graphene, crystal phases can be identified, each consisting of carbon atoms organized in a honeycomb lattice structure, with an orientation that differs from one domain to another. At the boundaries between domains, the three-fold coordination of the bond structure is preserved, but the honeycomb structure is discontinued by the presence of strings of five- and seven-fold rings.

Identifying different crystal phases and their orientations can be challenging. In our simulations, we employ a method called graph clustering to identify the phases and their orientations. This approach is sensitive enough to detect sub-phases with subtle differences in domain orientations. The local structural environment and orientation of each atom is determined using the PTM algorithm, then graph edge weights are initialized as $\exp(-d^{2}/3)$, where *d* is the misorientation in degrees between two neighboring atoms. Domains are built up by contracting edges using the Node Pair Sampling method of Bonald et al. [43]. In our simulations, two important parameters, the merge threshold and the minimum grain size, are set to 11 and 10, respectively, to achieve the best performance.

**Figure 6:**
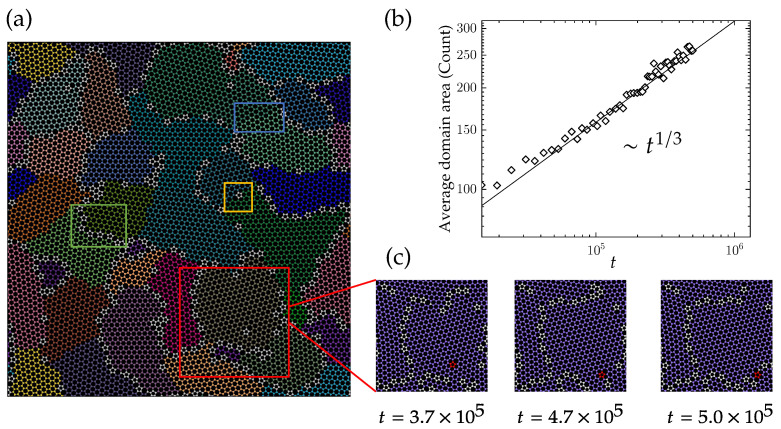
(**a**) A polycrystalline graphene with 9800 atoms; 36 crystal phases are identified by using the graph clustering algorithm. Green, yellow and blue boxes represent three different kinds of defect structures; details see in the text. (**b**) The average domain area changes in time, which approximately scales as a power law with a 1/3 exponent. (**c**) Movement of a defect island from the inside domain to thr grain boundary, showing the diffusive behavior of the defect.

Our simulations show that the dynamics of the domain structure in polycrystalline graphene are dominated by the motion of defects and domain boundaries. As shown in Figure 6a, 36 crystal phases (domains) are identified in a polycrystalline graphene consisting of 9800 atoms. Qualitatively, we observe various mechanisms that together constitute the dynamics of the domain structure. First, domain boundaries formed by strings of five and seven rings separate the domains (in the green box of Figure 6a). These domain boundaries are mobile, as is also observed experimentally using electron scanning microscopy. Second, isolated defects within the crystal domain exert a planar force on the adjacent lattices (in the yellow box of Figure 6a). Third, shear stress generated by grain boundaries on both sides of the domain shears it into two fragments (in the blue box of Figure 6a). During the domain growth process, the motion of defects can be classified into two scenarios. Some defects spontaneously disappear due to energy reduction, while others undergo diffusion motion. Figure 6c illustrates an example of defect diffusion, where a defect island located at the center of a domain moves to the adjacent continuous grain boundary after approximately 1.3 × 10${}^{5}$ Monte Carlo steps. Upon reaching the grain boundary, it cannot cross over to the crystal domain on the other side of the grain boundary.

We continue with a quantitative discussion of the evolution of the domain structure. The number of atoms in the domain is used as a representative measure of the domain area. In Figure 6b, the average domain size is plotted as a function of time. The square root of the average domain area exhibits a power-law increase with an exponent of 1/6. Given that the domains do not show a fractal structure, this is consistent with the decay exponent of defect density shown in Figure 3d.

At a first glance, the domain growth process in graphene resembles that of many other systems showing Ostwald ripening. A prototypical domain growth process is that in the Ising model [44]. With spin-flip (Glauber) dynamics, the theoretical framework is known as “Model A”, in which domains of aligned spins grow proportional to $t^{1/2}$. If the magnetization is locally conserved, as in spin-exchange (Kawasaki) dynamics, the theoretical framework is known as “Model B”, in which these domains grow proportional to $t^{1/3}$. In the case at hand, we do observe a growth exponent close to 1/3, but it is less clear that a local conservation law is active. There are a number of differences between the domain growth in graphene and the Ising model. For instance, the domains in graphene have a continuously varying orientation, rather than only “up” and “down”; additionally, long-ranged interactions might play a role, especially if buckling is allowed; and while some domain walls can easily move in some directions, the motion can be blocked in other directions. In future work, we hope to make a clearer connection between domain growth in graphene and the extensive literature on Ostwald ripening.

### 4.3. Domain Growth in Buckled Polycrystalline Graphene

The lowest-energy state of crystalline graphene in vacuum is a purely 2D structure. At finite temperature already, carbon atoms show out-of-plane displacements. Once structural defects are introduced, a free-floating layer of graphene shows even more structure in the out-of-plane direction. This buckling is suppressed significantly, but not completely, if the layer of graphene is placed on a substrate. For the current study on domain growth, the main effect of the substrate is the suppression of buckling. We therefore incorporate the main effect of the substrate by adding a harmonic confining energy term, defined as

(6) $$\begin{array}{c} E_{s}=K\sum_{i}^{N}z_{i}^{2}. \end{array}$$

**Figure 7:**
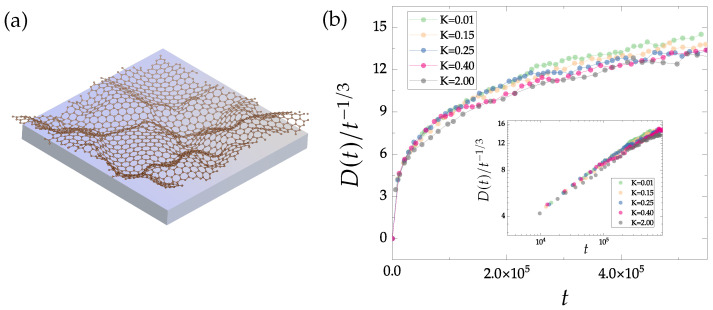
(**a**) A brief schematic diagram illustrating the growth of buckled polycrystalline graphene on a substrate. (**b**) Defect density of buckled polycrystalline graphene growing on various substrates divided by $t^{-1/3}$ (the decay rate in the flat case) over time. The inner figure is plotted on a double-logarithmic scale, demonstrating that the buckling of polycrystalline graphene slows down the crystallization rate. All samples are evolved starting from an initial configuration with 20% defect density. Since a well-crystallized sample leads to a higher buckling height Δz, which counteracts the suppression of substrate, the difference is weak for various values of *K* (eV Å${}^{-2}$).

Here, *N* is the number of atoms, $z_{i}$ is the normal-to-plane coordinate of the atom. Parameter *K* sets the strength of the interaction with the substrate. Tison et al. [45] have reported that the buckling resulting from defects and domain boundaries extends to typically 5 to 20 Å; according to our previous investigations, this corresponds to a range of *K* values between 0.05 and 0.3. Figure 7a displays a buckled polycrystalline graphene growing on a substrate, while Figure 7b focuses on the evolution of defect density in time. Specifically, the density of non-hexagonal rings is divided by $t^{-1/3}$ (the decay rate in the flat case) for various *K* values ranging from 0.01 to 2.00 eV Å${}^{-2}$.

Notably, our findings indicate that the buckling of polycrystalline graphene significantly slows down the domain growth process. As the value of *K* increases, $D\left(t\right)/t^{-1/3}$ tends to reach a constant value in a shorter time. The difference for various *K* is, however, weak, because the higher buckling height Δz resulting from crystallization counteracts the suppression of substrates. In conclusion, flatter graphene exhibits faster coarsening. This intriguing observation highlights the intricate interplay between buckling, substrate effects, and defect dynamics in the crystallization process of graphene.

## 5. Summary

In this paper, we employed a recently developed and extensively validated model to investigate the dynamics of domain growth in polycrystalline graphene. The dynamics consist of a sequence of proposed bond transpositions at random locations, accepted or rejected according to the Metropolis method. The technique enables access to much longer time scales compared to those of the molecular dynamics (MD) method. The studied domain growth process is performed under zero pressure, quenching the system from infinite temperature to approximately 3000 K. The radial distribution function shows that the spatial structures of our generated samples have good agreement with the ones in experiments at the same defect density.

Through simulations and analysis, the dynamics revealed underlying statistical mechanisms behind domain growth in polycrystalline graphene. Flat and buckled graphene were both investigated. For the flat case, we found that bond angles and bond lengths converged, respectively, towards $120^{\circ }$ and 1.42 Å as a function of time. The long-range disorder exhibited a gradual reduction, and the defect density, represented by the proportion of non-hexagonal rings, followed a power-law distribution with an exponent of −1/3 found from our simulations over time. In addition, the spatial correlation of lattice orientations statistically followed a stretched exponential form with less flat tail over times.

We identified different domains within polycrystalline graphene and delved into discussions regarding phase separation and defect diffusion motion. The average domain size exhibits a power-law increase with an exponent of 1/6 over times. We briefly compared the domain growth in polycrystalline graphene with the Ising dynamics. It was found that a similar growth exponent close to 1/3 was observed in the Kawasaki dynamics with a conserved magnetization density. However, the domain growth in polycrystalline graphene exhibits more complexity. Nevertheless, we believe that this correlation will provide some guidance for our future related research.

For the buckled case, we briefly investigated the evolution of buckled polycrystalline graphene on substrates. Our findings demonstrated that the undulating buckling of polycrystalline graphene led to a reduction in the crystallization rate.

Our work may provide crucial insights into the dynamics of polycrystalline graphene during crystallization processes, which is difficult to achieve in experiments and MD simulations. Our findings also contribute to a deeper understanding of the development of advanced materials and the optimization of graphene-based applications. Moreover, the observation of reduced crystallization rates in buckled polycrystalline graphene on substrates emphasizes the need for careful consideration of substrate effects in future graphene-related research.

## Data Availability

Data available on request to authors.
